# Multiplex Detection and SNP Genotyping in a Single Fluorescence Channel

**DOI:** 10.1371/journal.pone.0030340

**Published:** 2012-01-17

**Authors:** Guoliang Fu, Andrea Miles, Luke Alphey

**Affiliations:** 1 Oxitec Limited, Oxford, United Kingdom; 2 Department of Zoology, University of Oxford, Oxford, United Kingdom; St. Petersburg Pasteur Institute, Russian Federation

## Abstract

Probe-based PCR is widely used for SNP (single nucleotide polymorphism) genotyping and pathogen nucleic acid detection due to its simplicity, sensitivity and cost-effectiveness. However, the multiplex capability of hydrolysis probe-based PCR is normally limited to one target (pathogen or allele) per fluorescence channel. Current fluorescence PCR machines typically have 4–6 channels. We present a strategy permitting the multiplex detection of multiple targets in a single detection channel. The technique is named Multiplex Probe Amplification (MPA). Polymorphisms of the CYP2C9 gene (cytochrome P450, family 2, subfamily C, polypeptide 9, CYP2C9*2) and human papillomavirus sequences HPV16, 18, 31, 52 and 59 were chosen as model targets for testing MPA. The allele status of the CYP2C9*2 determined by MPA was entirely concordant with the reference TaqMan® SNP Genotyping Assays. The four HPV strain sequences could be independently detected in a single fluorescence detection channel. The results validate the multiplex capacity, the simplicity and accuracy of MPA for SNP genotyping and multiplex detection using different probes labeled with the same fluorophore. The technique offers a new way to multiplex in a single detection channel of a closed-tube PCR.

## Introduction

Recently, real-time PCR, which is carried out in a closed-tube format, has become a very important tool for detection and quantification of specific sequences in a sample [1]–[4]. Multiplex PCR, which uses multiple pairs of primers to amplify multiple target sequences simultaneously in a single PCR reaction, is likely to be a more efficient approach than standard single primer-pair PCR when multiple targets need to be detected [5]–[8].

Multiplex real-time PCR offers numerous advantages, including time savings, elimination of contamination risk, reduced reagent costs, increased throughput, conservation of precious sample material and reliable results [9]–[12]. However, current probe-based methods allow detection of only one target sequence per fluorescence channel. The multiplex fluorescent PCR is therefore limited by the availability of fluorescence dyes and channels; up to five or six targets, depending on particular PCR machines, can be detected simultaneously in the current platforms [5], [13].

We have developed a novel multiplex detection method (Multiplex Probe Amplification, MPA), which allows the detection of up to four targets in a single detection channel, using current standard instrumentation. As model targets against which to test this approach, we selected CYP2C9*2 SNP genotyping, and detecting and typing high-risk HPV sequences, each in a single closed-tube reaction. Variations in CYP2C9 gene may lead to differential responses and metabolism of numerous drugs, such as warfarin. Multiple-tube PCR, sequencing and microarray hybridisation techniques have been utilised for genotyping these SNPs [14], [15]. Human papillomavirus (HPV) is one of the most common causes of sexually transmitted disease (STD) worldwide; high-risk types of HPV can cause cervical cancer. Accurate molecular diagnosis is needed to inform patient management and follow-up after treatment [16]. In this study, two alleles of one SNP or four strains of a pathogen sequence were successfully genotyped in a single detection channel. This opens up the prospect of multiplex detection of far more targets in a single closed-tube reaction than the 4–6 currently possible with conventional PCR technology.

## Results

### Principles and Melting Profile of Probes

The principles of MPA are as follows: Firstly, probes labeled with the same fluorescence dyes can be distinguished if the probes themselves, independent of hybridising to target sequence, have different melting temperatures. Differential melting temperatures for free probes can be arbitrarily arranged by providing complementary oligonucleotide with sequences of varying length, base composition and/or complementarity to the target-hybridising oligonucleotide. Secondly, if a target is present, its corresponding probe is consumed during PCR amplification. Comparing melting profiles of the probes after the reaction reveals which probes have been consumed; this in turn indicates which targets are present in a sample.

**Figure 1:**
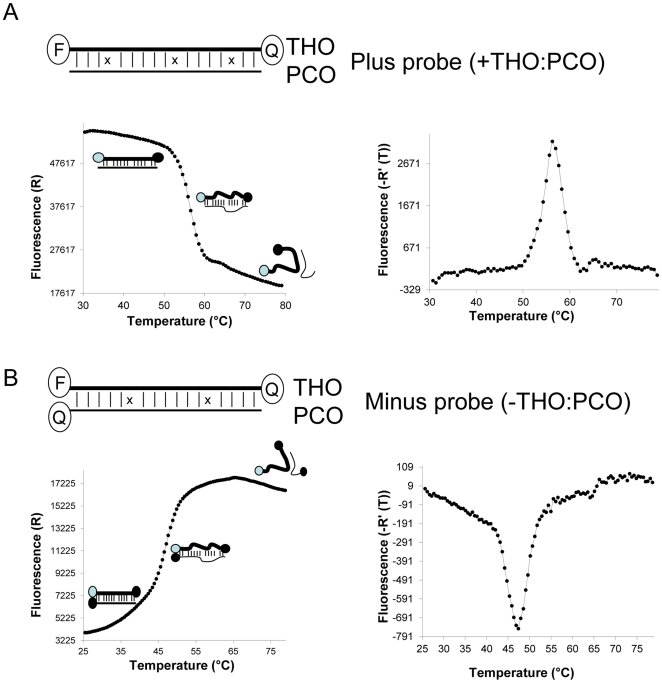
Nucleic acid probe design and its melting profile. (A) Probe consists of a target-hybridising oligonucleotide (THO) labeled with a Fluorophore at the 5′ end and a Quencher at the 3′end, and a partially complementary oligonucleotide (PCO) without a label. The hybrid of THO:PCO is named plus probe. The melting curve shows decreased fluorescence emission when temperature rises (left panel); the negative derivative plot of the emission reading versus temperature reveals a positive value of the melting peak (right panel). (B) Minus probe consists of a THO labeled with a Fluorophore at the 5′ end and a Quencher at the 3′end, and a PCO labeled with a Quencher at the 3′ end. The melting curve shows increased fluorescence emission when temperature rises (left panel); the negative derivative plot of the emission reading versus temperature reveals a negative value of the melting peak (right panel).

**Figure 2:**
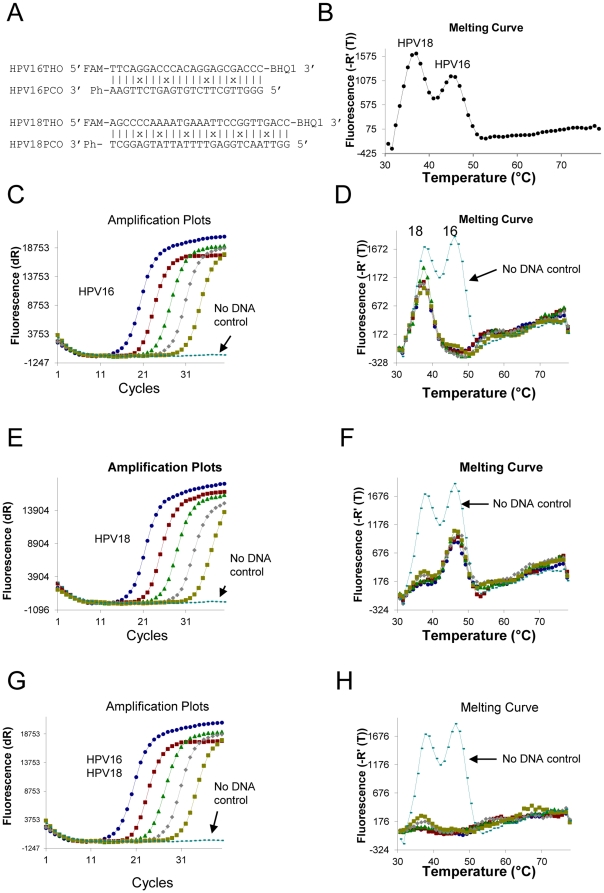
Design of HPV16 and HPV18 probes and the use of the probes for amplification and melting curve analysis. (A) Hybrids of THO:PCO are formed through hybridisation of THO and PCO. (B) Melting profile of the mix of HPV16 and HPV18 probes is plotted as the negative derivative of the emission reading versus temperature. (C) is the graphic presentation of the amplification plot of PCR on five 10-fold serial dilutions of HPV16 templates. (D) is the same reaction as (C) showing the melting curve. (E) is the graphic presentation of the amplification plot of PCR on five 10-fold serial dilutions of HPV18 templates. (F) is the same reaction as (E) showing the melting curve. (G) is the graphic presentation of the amplification plot of PCR on five 10-fold serial dilutions of the mix of HPV16 and HPV18 templates. (H) is the same reaction as (G) showing the melting curve.

For each target we designed a probe consisting of two oligonucleotides: a dual-labeled target-hybridising oligonucleotide (THO) and a partially complementary oligonucleotide (PCO). THO is equivalent to the hydrolysis probe in the traditional TaqMan PCR method. THO and PCO are capable of hybridising to each other, forming a partially double-stranded probe (Figure 1). By design, each probe has unique melting properties, which are characterised by its melting temperature (T_m_). THO, which is complementary to a target sequence, is labeled with a fluorophore at the 5′ end and a quencher at the 3′ end. PCO has its 3′ end blocked by modifying the 3′ end, for example, by attaching a label or a phosphate group, so that it cannot be extended. If the 3′ end of PCO comprises a moiety which is not a quencher (Figure 1A and 2A), fluorescence emission is at the highest amount when THO and PCO are hybridised. The fluorescence emission is decreasing when the temperature is increasing, which opens up the duplex (Figure 1A, the left panel). The reason for this phenomenon is that a linear single-stranded dual-labeled oligonucleotide in solution behaves like a random coil: its two ends occasionally come close to one another, resulting in a measurable change in energy transfer. However, when the dual-labeled oligonucleotide binds to its template, the labeled oligonucleotide/template hybrid forces the two ends of the probe apart, disrupting the interaction between the two terminal labels, and thus causing a fluorescence emission change. The derivative melting plot shows a positive value (Figure 1A, the right panel), thus this type of probe is termed ‘plus probe’ (+THO:PCO).

If PCO is labeled with a quencher at the 3′ end (Figure 1B), fluorescence emission is decreased by hybridisation of THO and PCO and increases when the temperature increases to denature the duplex (Figure 1B, left panel). This is because formation of the THO:PCO hybrid brings the quencher and the fluorophore into close proximity, efficiently quenching the fluorescent signal. The derivative melting plot shows a negative value (Figure 1B, right panel), thus this type of probe is termed ‘minus probe’ (-THO:PCO).

In a multiplex PCR reaction of this system, there are at least two probes for two different targets, which are labeled with the same fluorophore. To distinguish them, each probe is designed to have a unique T_m_ which can be recognised in a melting curve analysis. For example, we designed two probes: one for HPV16 sequence and one for HPV18 sequence, which are both plus probes (Figure 2). HPV16 probe has a T_m_ of 46°C and HPV18 probe has a T_m_ of 37°C. These clearly show two peaks in the melting curve (Figure 2b).

The target-hybridising oligonucleotide (THO) capable of hybridising to the target sequence can be consumed during amplification. In the absence of a target, THO is not consumed, thus remains at the same concentration throughout the reaction. The melting profile of the unconsumed probe does not change. In the presence of a target, the THO preferentially hybridises to the target sequences rather than to the PCO, because T_m_ of THO:target is higher than the T_m_ of THO:PCO. As a result, THO is consumed and its concentration decreases. The melting peak of the consumed probe is reduced or disappears in the melting curve analysis.

The key feature of consumption of THO could be achieved by any of several methods. For example, THO might be incorporated into a PCR product, where THO acts as a primer. In this study, we designed the THO to work as a hydrolysis probe, which is hydrolysed during the amplification [17]. The degradation of THO, like ordinary probe-based real-time PCR, results in the increase of fluorescence signal, which can be monitored during PCR. In the mean time, during the melting curve analysis, the degradation of THO results in the decrease or disappearance of its signature melting peak, indicating that the corresponding probe is being consumed, which in turn shows which target is amplified.

### Plus Probes Detecting HPV16 and HPV18

To test this system, we designed probes and primers for detecting the two most virulent and high risk HPV strains, HPV16 and HPV18, in a single fluorescent detection channel in a real-time PCR machine. Two plus probes were designed, targeting the E6/E7 region of the HPV genome sequence (Figure 2A). Real-time PCR was performed to test the design. Probe hybrids THO:PCO were pre-tested in a melting curve analysis to determine the fluorescence levels, so that when they were mixed together, they had similar heights of melting peaks (Figure 2B). Five 10-fold serial dilutions (10^6^ to 10^2^ copies) of template plasmids containing the target HPV sequences, and a no DNA negative control, were PCR amplified, and fluorescence emission was collected in the FAM channel. Typical real-time PCR amplification plots for each series of template dilutions are shown in Figure 2 (panel C – HPV16, panel E – HPV18, panel G – HPV16+HPV18). Melting profiles were obtained after amplification. Compared with the negative control, when the target HPV16 is present, the melting peak at 46°C in the melting curve has disappeared (Figure 2D). When the target HPV18 is present, the melting peak at 37°C has disappeared (Figure 2F). When both targets HPV16 and HPV18 are present, the two melting peaks have both disappeared (Figure 2H). The results clearly demonstrate that HPV16 or HPV18 or both HPV16+HPV18 can be detected individually and distinguished, even in the same detection channel.

### Plus Probes Genotyping SNP CYP2C9*2

**Figure 3:**
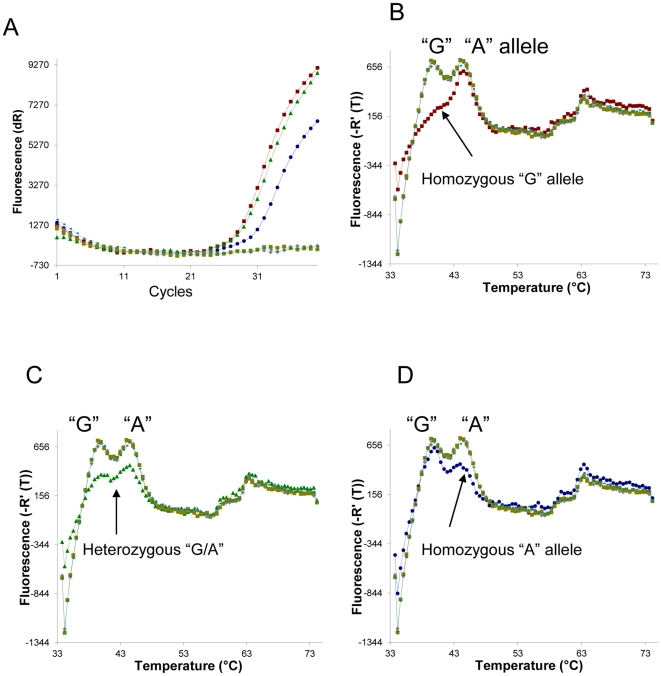
Plus probes genotyping SNP CYP2C9*2. (A) is the graphic presentation of the amplification plot of PCR for genotyping SNP CYP2C9*2 on three genomic DNA samples and three negative controls. (B) is melting curves showing one sample is homozygous for G allele. (C) is melting curves showing one sample is heterozygous. (D) is melting curves showing one sample is homozygous for A allele.

To test if this method can be used for SNP genotyping, we designed probes and primers for genotyping SNP CYP2C9*2 in a single fluorescent detection channel in a real-time PCR machine. Two plus probes were designed, one corresponds to the A allele (c.430T) and one corresponds to the G allele (c.430C). “A” allele probe has a T_m_ of 44°C; “G” allele probe has a T_m_ of 39°C. If a particular SNP is present in a sample, its corresponding probe is hydrolysed by the 5′ nuclease activity of *Taq* polymerase during amplification, resulting in an increase in fluorescence emission. We genotyped 25 samples using this design. Figure 3 shows amplification plots (Figure 3A) and melting curves (Figure 3B, 3C and 3D). The melting curve of a homozygous sample of “G” allele along with no amplification controls is shown in Figure 3B. A heterozygous sample along with no amplification controls is shown in Figure 3C. A homozygous of “A” allele along with the no amplification controls is shown in Figure 3D. This result is 100% in concordance with TaqMan® SNP Genotyping Assays from Applied Biosystems.

### Minus Probes Detecting Four HPV Sequences

**Figure 4:**
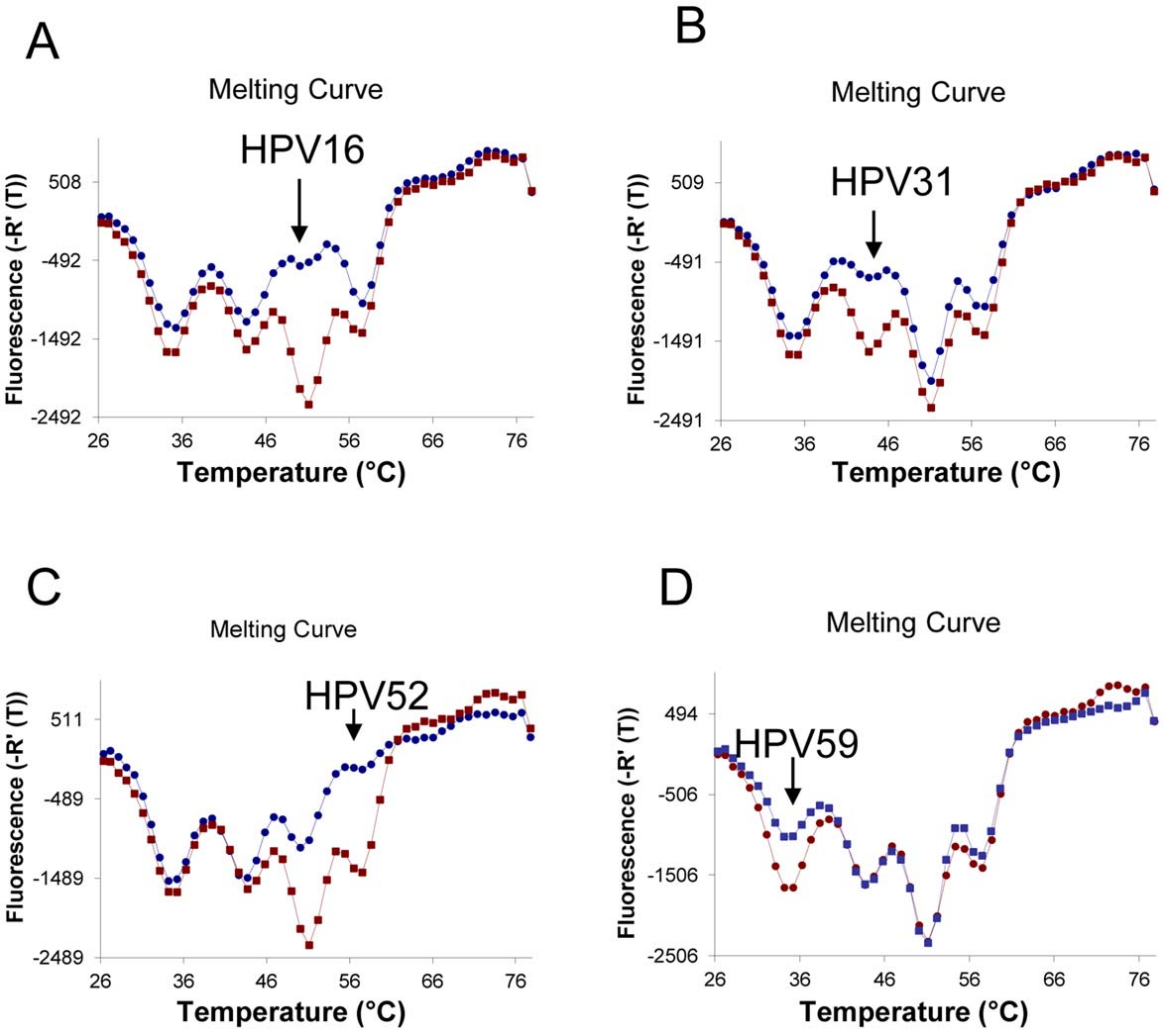
Melting curve analysis of the amplification reactions of various HPV targets. When a HPV target is present in a reaction, its corresponding probe is consumed and, in comparison with the negative control, the appropriate melting peak in the melting profile is decreased or has disappeared. The negative control melting curve is marked in red; the target melting curve is marked in blue. (A) HPV16 is present. (B) HPV31 is present. (C) HPV52 is present. (D) HPV59 is present.

To test the approach further, with minus probes, different target sequences and more independent targets per channel, we designed four minus probes labeled with FAM dye for HPV16, HPV31, HPV52 and HPV59 sequences. The probes' THOs are complementary to the conserved L1 region of the HPV genome sequences. HPV59 probe has a T_m_ of 35°C; HPV31 probe has a T_m_ of 43°C; HPV16 probe has a T_m_ of 51°C; and HPV52 probe has a T_m_ of 57°C. After amplification, melting profiles were performed. Compared with the negative control, each individual HPV target sequences can be correctly distinguished in the FAM detection channel (Figure 4A–D).

### Sensitivity of the assays on genomic DNA

The limit of detection of the HPV assays using plus and minus probes was evaluated in a real-time PCR format. HeLa cell line genomic DNA containing HPV16 and SiHa cell line genomic DNA containing HPV18 were serially diluted in 10-fold increments. Real-time PCR assays were performed on the dilution series and negative controls; all were performed in duplicate. The HPV negative K562 cell line gDNA control and water control were all negative. The HeLa cell genome used contains 10 to 50 copies of HPV18 DNA, whereas the SiHa cell genome contains a single integrated copy of HPV16 [18]. A sensitivity of 0.03ng of gDNA was determined for both plus and minus probe assays. As 0.03ng of human genomic DNA is about 10 genome equivalents, this indicates that the assays can detect at least 10 copies of HPV16 DNA and 100–500 copies of HPV18 DNA in the background of human genomic DNA.

## Discussion

Compared with traditional hydrolysis probe-based PCR, this MPA method comprises an extra oligonucleotide, which is capable of hybridising to the labeled oligonucleotide. Since the extra oligonucleotide hybridises to the labeled oligonucleotide at a temperature lower than the annealing temperature, it does not affect the PCR process. In other words, in terms of PCR amplification there is no difference between this multiplex method and the traditional hydrolysis probe PCR. Therefore, this new method inherits all the benefits of the traditional hydrolysis probe-based PCR, but with an extra benefit of being able to multiplex using probes labeled with the same fluorophore and detection in a single fluorescent channel.

To assess the scope and flexibility of MPA, we have also performed experiments using additional fluorophores rather than FAM, using plus and minus probes together, using minus probes and a single-stranded probe, and even detecting all 14 high-risk HPV strain sequences in a single-closed tube reaction. In one experiment, four minus probes labeled with HEX dye were designed, detecting HPV18, HPV39, HPV58 and HPV68 sequences (Figure S1A–D). In another experiment, a combination of plus and minus probes was tested for detecting HPV33, HPV45 and HPV35 sequences (Figure S1E–G). A combination of minus probe and single-stranded probe was also tested. Two minus probes labeled with Cy5 dye for HPV56 and HPV66 sequences, and one single stranded probe labeled with the same Cy5 dye for HPV51 sequence were included in a PCR reaction (Figure S1I–J). These experiments demonstrated that an individual HPV sequence can be distinguished by using a combination of multiple minus probes, plus probes and single-stranded probes.

When analysing the melting profiles, we have noticed that peaks adjacent to the ‘true’ peak may also show a modest reduction in peak height in minus probe assays for some HPV targets (Figure 4C, S1A–B). This “shrunk neighbouring peaks phenomenon” could potentially lead to the erroneous conclusion that two or more targets were present in the original template. In follow-up experiments, using different PCR conditions and master mix, we found this phenomenon to be assay-specific but highly reproducible. Though the origin of this effect remains unclear, it does not affect the diagnostic specificity and accuracy of the assay – the signature melting profile for single HPV target is clearly different from the melting profiles for double or triple HPV targets. One always can judge whether it is single, double or triple HPV infection by comparing with reference melting profiles generated from single and double target standards.

Recently, a novel method named Multicolor Combinatorial Probe Coding (MCPC) was described for increasing the target numbers of real-time PCR detection in one reaction [19]. This strategy uses differently coloured fluorophores in various combinations to label individual probes. It successfully detected up to 15 targets in a single reaction. However, one of the major limitations of MCPC is that it is not suitable for simultaneous detection of multiple targets in one reaction, as the colour overlaps between singly labelled, doubly labelled, and multiply labelled probes complicate the MCPC profiles. Fortunately MPA, with its different and much simpler labelling strategy, does not suffer from this limitation, indeed in our HPV assay we routinely detected multiple targets in one reaction (Figure 2 and data not shown).

Our results demonstrate that it is possible to overcome the current one-channel-one-target limitation. By this method a greater number of target sequences can be analysed in a single closed tube by designing sets of probes that hybridise to different target sequences and have different melting temperatures. If a target sequence is present, its corresponding probe is consumed. The target can then be determined based on the comparison of the melting profiles of the probes before and after the reactions. Groups of probes in a set can be attached with the same label, allowing for independent monitoring of multiple targets at a single emission wavelength. Our method provides a significant improvement over the current closed-tube multiplex PCR technology, allowing for a 2–4 fold increase in the number of targets being analysed in the current instruments.

## Materials and Methods

### Cell line genomic DNA and plasmid DNA

The HPV18 positive cervical HeLa cell DNA was purchased from New England Biolabs; the HPV16 positive cervical SiHa cell line was purchased from Abgent; the HPV negative K562 cell line DNA was purchased from Promega. Genomic DNA was extracted using GeneJET™ Genomic DNA Purification Kit from Fermentas according to manufacturer's instruction. Plasmid DNAs containing HPV sequences were synthesised by GeneArt. Serial 10-fold dilutions were undertaken to produce a titration series representing from 30ng to 0.03ng/µl for genomic DNA, and 10^6^ to 10^2^ copies/µl for plasmid DNA.

### Probe Design and Oligonucleotide Synthesis

Two plus probes were designed, targeting the E6/E7 region of the HPV genome sequence. The target-hybridising oligonucleotides (THOs) were synthesised with a FAM label at the 5′ end and a BHQ1 at the 3′ end. The nucleotide sequence for HPV16 THO is: 5′ TTCAGGACCCACAGGAGCGACCC 3′. The nucleotide sequence for HPV18 THO is: 5′ AGCCCCAAAATGAAATTCCGGTTGACC 3′. Partially complementary oligonucleotides (PCOs) were synthesised with the same length as the THOs and were attached with a phosphate group at the 3′ end. The nucleotide sequence for HPV16 PCO is: 5′ GGGTTGCTTCTGTGAGTCTTGAA 3′. The nucleotide sequence for HPV18 PCO is: 5′ GGTTAACTGGAGTTTTATTATGAGGCT 3′. A THO and PCO were combined at a ratio of 1∶2 to form a partially double-stranded nucleic acid probe. Forward and reverse primers were designed to be upstream and downstream of the probe-binding region. The sequences for HPV16 forward and reverse primers are 5′ AGACATTTTATGCACCAAAAGAGAACT 3′ and 5′ TCTGTGCATAACTGTGGTAACTTTCTG 3′, respectively. The sequences for HPV18 forward and reverse primers are 5′ GTATGCATGGACCTAAGGCAACA 3′ and 5′ TCGCTTAATTGCTCGTGACATAGA 3′, respectively. Oligonucleotides were synthesized by Eurogentec.

Two plus probes were designed, targeting the SNP CYP2C9*2. The target-hybridising oligonucleotides (THOs) were synthesised with FAM at the 5′ end and BHQ1 at the 3′ end. The nucleotide sequence for the “G” allele probe is: 5′ CTTGAACACGGTCCTCAATGC 3′. The nucleotide sequence for the “A” allele probe is: 5′ TCTTGAACACAGTCCTCAATGCT 3′. The partially complementary oligonucleotide (PCO) was the same for both allele probes and was attached with a phosphate group at the 3′ end. The nucleotide sequence of the PCO is: 5′ GCATTAAGGACTGTGTCCAAGA 3′. Forward and reverse primers were designed to be upstream and downstream of the probe-binding region. The sequences for forward and reverse primers are 5′ TAAG GTCAGTGATA TGGAGTAGG 3′ and 5′ GAATTGTTTTCAGCAATGGAAAGAA 3′, respectively.

Consensus primers BSGP5+/BSGP6+ targeting the highly conserved L1 region that have the potential to detect all mucosal HPV types were used in PCR detection with minus probes. PCR amplification by BSGP5+/BSGP6+ has been described [20]. Four minus probes labeled with FAM dye were designed, targeting the conserved L1 region of the HPV genome sequences of HPV16, HPV31, HPV52 and HPV59. The target-hybridising oligonucleotides (THOs) were synthesized with FAM at the 5′ end and BHQ1 at the 3′ end. The nucleotide sequence for HPV16 probe is: 5′ CTGCCATATCTACTTCAGAAACTACATATAA 3′. The nucleotide sequence for HPV31 probe is: 5′ CTGCAATTGCAAACAGTGATACTACATTTAA 3′. The nucleotide sequence for HPV52 probe is: 5′ CTGAGGTTAAAAAGGAAAGCACATATAAAAATG 3′. The nucleotide sequence for HPV59 probe is: 5′ CTTCTACTACTTCTTCTATTCCTAATGTATAC 3′. The partially complementary oligonucleotide (PCO) was the same for HPV16 and HPV31 probes and was attached with a Dabcyl group at the 3′ end, having a sequence 5′ TTATATGTAGTATCTGAGTTAGCTATGGCAG 3′. The PCO for the HPV52 probe was attached with a BHQ-1 group at the 3′ end, having a sequence 5′ CATTTTTATATGTGCTGTCCTTAGTAACTTCAG 3′. The PCO for the HPV59 probe was attached with a BHQ-1 group at the 3′ end, having a sequence 5′ GTATATATTTGGTACAGGAGACTCTGTAGAAG 3′.

### Real-time PCR and Melting Profile Analysis

PCR reactions in a final volume of 25 µl consist of two equal amounts of mix: 12.5 µl of 2× FastStart Universal Probe master (Rox) (Roche Diagnostics Gmbh, Mannheim Germany) and 12.5 µl primer/probe mix. Primer/probe mix was created as follows: the primers and probes were mixed to a final concentration of 0.4 µM of probes and 0.6 µM of primers, and 1 µl of various amount of target templates were added. Amplification reactions and melting profiles were performed in a Stratagene real-time PCR MX3005P system. The thermal profile was: 95°C for 9 min 30 sec; 40 cycles of 95°C for 20 sec and 60°C for 60 sec. Fluorescence measurements were recorded during the read steps at 60°C. Post-amplification melting profile had the following conditions: after the last cycle of PCR, heat at 95°C for 10 sec, cool to 30°C and hold for 30 sec, then slowly increase the temperature to 80°C. The fluorescence emission data is continually collected during the rising temperatures. The negative derivative of the emission reading, with respect to temperature, is plotted against the temperature to form melting curves, and the peak of the curve corresponds to the T_m_ of the probe.

## Supporting Information

Figure S1 **Melting curve analysis of the amplification reactions of various HPV targets.**
(DOCX)
