# Supplementary material for: Multiplex Detection and SNP Genotyping in a Single Fluorescence Channel
Source: PLoS One. 2012 Jan 17;7(1):e30340. doi: 10.1371/journal.pone.0030340 (PMC3260291; doi:10.1371/journal.pone.0030340)
Supplement: Figure S1 — Melting curve analysis of the amplification reactions of various HPV targets. (DOCX) [file pone.0030340.s001.docx]

HPV56

HPV66

I

J

**Figure. S1 Melting curve analysis of the amplification reactions of various HPV targets.** Four minus probes labeled with HEX dye were designed, targeting the conserved L1 region of the HPV genome sequences, which detect HPV18, HPV39, HPV58 and HPV68 sequences. Figure S1A-D show that these four HPV sequences can be correctly genotyped. A combination of plus and minus probes were tested for detecting HPV sequences (E, F and G). Two minus probes labeled with Texas Red dye, targeting the L1 region of HPV33 and HPV45 sequences, and one plus probe labeled with the same Texas Red dye, targeting the L1 region of HPV35 sequence were included in a PCR reaction. After PCR amplification, a melting curve analysis was performed. Compared with the no DNA control, in the presence of each target, the corresponding melting peak either reduced or disappeared (Figure S1E-G). A combination of minus probe and single-stranded probe were also tested for detecting three HPV strains (H, I and J). Two minus probes labeled with Cy5 dye, targeting the L1 region of HPV56 and HPV66 sequences, and one single stranded probe labeled with the same Cy5 dye, targeting the L1 region of HPV51 sequence were included in a PCR reaction. After PCR amplification, a melting curve analysis was performed. Compared with the no DNA control, in the presence of HPV56 or HPV66, the corresponding melting peak either reduced or disappeared (Figure S1I-J). However, when the target HPV51 template was present, the melting peaks for HPV56 and HPV66 did not change, but the whole fluorescence signal increased (Figure S1H). The increase of the whole fluorescence signal is reflected by proportionally lifting up its melting curve in comparison with the negative control. The amplification plots also show a normal amplification curve, which is due to the presence of the HPV51 sequence. The negative control melting curve is marked in red; the target melting curve is marked in blue. (A) HPV18 is present. (B) HPV39 is present. (C) HPV58 is present. (D) HPV68 is present. (E) HPV33 is present. (F) HPV35 is present. (G) HPV45 is present. (H) HPV51 is present. (I) HPV56 is present. (J) HPV66 is present.
